# Genotoxicity Integration into Bioprocess Optimization Reveals Progressive DNA Damage During Bioreactor Expansion of Adipose-Derived Stem Cells

**DOI:** 10.3390/ijms27114795

**Published:** 2026-05-26

**Authors:** Vinícius Augusto Simão, Rafaela Choi Peng So, Jaci Leme, Rafael Guilen de Oliveira, Gabriel Adan Araújo Leite, Luiz Gustavo de Almeida Chuffa, Aldo Tonso, João Tadeu Ribeiro-Paes

**Affiliations:** 1 Department of Biotechnology, School of Sciences, Humanities, and Languages, São Paulo State University (UNESP), Assis 19806-900, São Paulo, Brazil; 2 Laboratory of Viral Biotechnology, Center for Development and Innovation, Butantan Institute, São Paulo 05503-900, São Paulo, Brazil; 3 Department of Cell Biology, Embryology and Genetics, Center for Biological Sciences, Federal University of Santa Catarina (UFSC), Florianópolis 88040-900, Santa Catarina, Brazil; 4 Department of Structural and Functional Biology, Institute of Biosciences, São Paulo State University (UNESP), Botucatu 18618-689, São Paulo, Brazil; 5 Department of Chemical Engineering, Polytechnic School, University of São Paulo (USP), São Paulo 05508-010, São Paulo, Brazil

**Keywords:** genotoxicity, DNA damage, genomic integrity, comet assay, micronucleus test, human mesenchymal stromal cells, adipose-derived stem cells, bioreactor, microcarrier culture, metabolic profiling

## Abstract

Mesenchymal stromal cells derived from adipose tissue (ASCs) are widely used in regenerative medicine, requiring scalable expansion strategies that preserve both cellular function and biological quality. However, current bioprocess optimization approaches are primarily guided by proliferation and phenotypic stability, often overlooking genomic integrity as a critical attribute. In this study, we developed a stirred-tank bioreactor system for ASC expansion on microcarriers and applied a genotoxicity-informed optimization strategy by integrating growth kinetics, metabolic profiling, and DNA damage assessment across multiple operational conditions (B_1_–B_5_), including variations in dissolved oxygen, agitation, inoculum density, and medium renewal. Optimized culture conditions (B5) enabled high cell productivity within a reduced cultivation period (9 days), while maintaining high viability (>90%), mesenchymal immunophenotype, and differentiation capacity. Distinct metabolic profiles were associated with enhanced proliferation, with increased glycolytic activity observed under optimized conditions. Despite these favorable outcomes, genotoxic analyses revealed a progressive, time-dependent accumulation of DNA damage and increased micronucleus frequency during expansion. Notably, these alterations did not impair cell proliferation, phenotype, or differentiation potential, indicating that conventional optimization metrics may not fully capture underlying genomic changes. Collectively, our findings demonstrate that bioprocess optimization based solely on classical performance parameters may overlook relevant biological alterations. By incorporating genotoxic endpoints into the evaluation framework, this study provides a refined approach for assessing large-scale stem cell expansion and contributes to improving the robustness and reliability of biomanufacturing strategies for therapeutic applications.

## 1. Introduction

Mesenchymal stromal cells (MSCs) are a non-hematopoietic, fibroblast-like population characterized by plastic adherence. According to the International Society for Cellular Therapy (ISCT), mesenchymal stem cells must express CD105^+^/CD73^+^/CD90^+^, lack hematopoietic markers, and demonstrate osteogenic, chondrogenic, and adipogenic differentiation capacity [1]. Although first described in bone marrow, MSCs have since been isolated from multiple tissues, with adipose tissue standing out due to its minimally invasive collection, high availability, and a colony-forming unit frequency up to 500-fold higher than that of bone marrow [2,3]. Adipose-derived stem cells (ASCs) also exhibit high viability and phenotypic stability after expansion, supporting their increasing use in regenerative medicine and clinical applications [4,5,6,7,8,9,10,11].

For clinical-scale applications, MSC expansion requires millions of cells per dose, making traditional 2D static culture in T-flasks inefficient due to limited surface area, extensive handling, and suboptimal control of physicochemical parameters [12,13,14]. To address these limitations, 3D suspension culture using microcarriers (MCs) in stirred bioreactors has emerged as a scalable alternative for the proliferation of adherent MSCs [15,16]. Among the spherical beads, macroporous MCs such as Cultispher-S^®^ provide a high surface-to-volume ratio and partial protection against hydrodynamic stress, thereby enhancing cell attachment and proliferation [17,18].

Stirred-tank bioreactors further enhance scalability by enabling homogeneous mixing, real-time monitoring, and automated control of key parameters such as dissolved oxygen, pH, temperature, and gas exchange [19,20,21]. However, despite these advantages, standardized and optimized cultivation strategies for ASCs remain limited. Most studies focus on individual parameters—such as oxygen levels, agitation, or microcarrier type—without integrating multiple operational variables into a unified optimization framework [22,23,24,25,26,27,28,29,30,31]. Thus, comprehensive studies integrating operational culture parameters, initial seeding density, and evaluation of MC aggregates are still needed to establish robust, optimized bioprocesses for ASCs expansion and production of the intended cellular products.

Importantly, current bioprocess optimization strategies are predominantly guided by cell yield, viability, and phenotypic stability. While these parameters are essential, they may not fully reflect underlying biological changes occurring during large-scale expansion. In particular, genomic integrity represents a critical quality attribute for stem cell-based therapies, as DNA damage accumulated during in vitro expansion may have long-term implications for safety and functionality [32,33,34]. Thus, beyond standard physicochemical analyses, implementing tools to ensure and monitor the genetic stability of MSC cultures is essential to guarantee the safety of cell-based therapies.

Genotoxicity assays such as the comet assay and the micronucleus test are widely recognized for their sensitivity in detecting DNA damage and chromosomal instability [35,36,37]. Despite their relevance, these approaches are rarely incorporated into bioprocess evaluation, and most studies assessing MSC expansion do not consider genomic integrity alongside proliferation performance. As a result, potential discrepancies between cellular expansion metrics and underlying genomic alterations remain largely unexplored. Existing studies primarily examine cellular senescence related to passage number or donor variability [38,39,40,41,42], the impact of neuronal differentiation media [34] or chemical compounds [43] on MSC genomic stability, the tumorigenic potential of DNA-damaged cells [44], pH effects [45], and optimization of micronucleus induction protocols [46]. This gap suggests that current optimization paradigms may overlook biologically relevant changes that are not captured by conventional performance indicators. Therefore, integrating genotoxic endpoints into bioprocess evaluation may provide a more comprehensive understanding of stem cell expansion dynamics.

In this context, the present study developed a stirred-tank bioreactor system for ASC expansion that integrates growth kinetics, metabolic profiling, and genotoxicity assessment across multiple operational conditions. This approach enables the evaluation of cell expansion performance alongside genomic integrity, providing new insights into the relationship between proliferation and DNA stability during large-scale stem cell cultivation.

## 2. Results

### 2.1. Balanced Oxygen Supply and Reduced Agitation Drive Optimal ASC Expansion in Bioreactors

To establish large-scale cultivation of ASCs in a bioreactor, three preliminary single-run experiments were conducted to assess the influence of agitation speed (rpm), initial inoculum concentration (cells/mL), and dissolved oxygen (DO) percentage on cell yield over 21 days of cultivation. Each experimental condition was performed sequentially with targeted adjustments to key parameters (Table 1). At the end of cultivation, the experiments were compared based on growth kinetics and fold-increase (*FI*) to determine the setup yielding the highest cell productivity. In parallel, additional analyses were performed to evaluate metabolic activity, differentiation potential, and genomic integrity, including DNA fragmentation assessed by the comet assay. This integrated approach enabled the assessment of cell expansion performance alongside underlying biological changes occurring during bioreactor cultivation.

**Figure 1:**
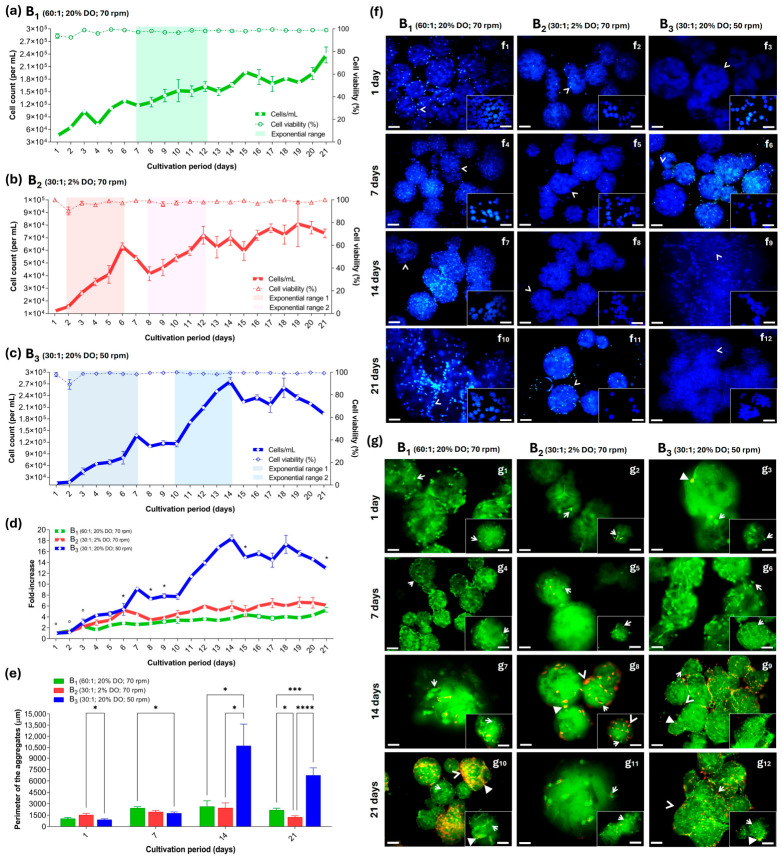
Growth kinetics, aggregate formation, and viability of ASCs cultured on MCs under different bioreactor conditions. (**a**–**c**) Cell growth curves showing cell concentration (viable adherent cells/mL) and viability of adhered cells (%) over 21 days under three culture conditions: B_1_ (60:1; 20% DO; 70 rpm) (**a**), B_2_ (30:1; 2% DO; 70 rpm) (**b**), and B_3_ (30:1; 20% DO; 50 rpm) (**c**). Shaded areas indicate the exponential growth phases. (**d**) Fold increase relative to the extent to which the culture proliferated over time for the three experimental conditions (B_1_, B_2_, and B_3_). ° *FI* values did not differ statistically from each other (*p* > 0.05); * *FI* were similar between specific conditions (B_2_ and B_3_ at day 6; B_1_ and B_2_ at days 8, 9, 15, and 21); *FI* lacking a superscript symbol indicated a statistically significant difference (*p* < 0.05) between all culture conditions. (**e**) Quantification of aggregate size, expressed as the perimeter (µm), at different cultivation times (days 1, 7, 14, and 21). Distinct asterisks indicate statistically significant differences between groups: *p* < 0.05 (*); *p* < 0.001 (***); *p* < 0.0001 (****). (**f**) Representative fluorescence microscopy images (DAPI staining, blue) showing cell distribution (thin arrowheads) and aggregate organization under the three culture conditions (B_1_–B_3_) at 1 (**f_1_**–**f_3_**), 7 (**f_4_**–**f_6_**), 14 (**f_7_**–**f_9_**), and 21 (**f_10_**–**f_12_**) days. Insets highlight MC distribution within aggregates. (**g**) Representative AO/EB staining images showing cell viability within MCs and aggregates at 1 (**g_1_**–**g_3_**), 7 (**g_4_**–**g_6_**), 14 (**g_7_**–**g_9_**), and 21 (**g_10_**–**g_12_**) days. Viable cells fluoresce green (thin arrows), apoptotic cells (thick arrows) fluoresce yellow, and cells with compromised membrane integrity fluoresce orange/red (thin arrowheads). Viable cells also showed lysosomes throughout the cytoplasm, colored in red (thin arrows). Scale bars: 50 µm ((**f_3_**,**f_9_**,**f_10_**,**g_1_**–**g_3_**,**g_5_**–**g_8_**,**g_11_**), and insets (**g_1_**–**g_12_**)), 100 µm (**f_1_**,**f_2_**,**f_4_**–**f_8_**,**f_11_**,**f_12_**,**g_4_**,**g_9_**,**g_10_**,**g_12_**), and 600 µm (insets (**f_1_**–**f_12_**)).

Figure 1a–c show the daily viable cell concentration (cells/mL) and total viability of MC-adherent ASC cultures under conditions B_1_, B_2_, and B_3_, respectively, while Figure 1d presents the comparative *FI* values throughout the cultivation period. Growth kinetic parameters derived from each growth profile are summarized in Table 2.

Although B_1_ resulted in the highest final cell concentration (2.4 × 10^5^ cells/mL) (Figure 1a), this outcome is attributable to its higher initial inoculum (60:1; 4.8 × 10^4^ cells/mL), which was twice that used in B_2_ and B_3_ experiments (30:1; 2.4 × 10^4^ cells/mL) (Figure 1b,c). However, this increased initial density did not translate into higher productivity, as the maximum *FI* of B_1_ was 5.3 at 21 days, whereas B_2_ reached 6.7 at 19 days and B_3_ achieved 18.4 at 14 days (Figure 1d). Despite starting with the same inoculum concentration, B_2_ (2% DO and 70 rpm) exhibited lower performance compared to B_3_, as evidenced by its reduced final cell concentration (7.3 × 10^4^ vs. 1.9 × 10^5^ cells/mL in B_3_) (Figure 1b) and significantly lower *FI* values (*p* < 0.05) (Figure 1d). Together, these results indicate that the combination of adequate oxygen availability (20% DO) and lower agitation (50 rpm), rather than inoculum density alone, was critical for maximizing cell expansion, as demonstrated by the superior growth performance observed in B_3_.

Notably, both B_2_ and B_3_ experiments showed two distinct exponential growth phases over the 21-day culture period (Figure 1b,d). Accordingly, both phases were analyzed in terms of kinetic parameters derived from the linearization of each exponential growth phase (Table 2). Despite its lower final cell density, B_2_ showed the second shortest doubling time (51.3 h) during its first exponential growth phase (days 2–6) (Figure 1b; Table 2), which was markedly shorter than that observed in its second exponential phase (123.7 h). In contrast, the superior performance of B_3_ was supported by the highest maximum specific growth rate (0.0144 h^−1^), corresponding to the shortest doubling time (48.1 h) during its first exponential phase among all bioreactor conditions. Furthermore, maximum cell productivity in B_3_ was not only higher but was also reached earlier (day 14), achieving the highest value observed (751.49 cells/mL/h) (Table 2).

The density of ASCs adhered to the MCs was monitored at days 1, 7, 14, and 21 days of cultivation using DAPI fluorescent staining under each experimental condition (B_1_, B_2_, and B_3_). As shown in Figure 1f, there was a progressive increase in cellular coverage on the porous MCs over the course of the culture. In B_1_, a higher number of adhered cells was observed at 24 h, which is consistent with its higher initial inoculum density (Figure 1(f_1_)). In contrast, B_2_ exhibited minimal changes in confluence throughout the entire cultivation period (Figure 1(f_2_,f_5_,f_8_,f_11_)). In B_3_, although the initial number of adhered cells was low (Figure 1(f_3_)), the culture underwent rapid proliferation. By 14 days—coinciding with the peak in cell concentration (Figure 1c)—large clusters of MCs bound together by a high density of adhered cells were already evident (Figure 1e,(f_9_)), with little variation observed thereafter up to 21 days (Figure 1(f_12_)). At the end of the culture, a reduction in aggregate perimeter was observed across all experimental conditions (B_1_ to B_3_) (Figure 1e). Notably, B_3_ exhibited the largest aggregate perimeters from 14 days onward, showing significant differences when compared to B_1_ and B_2_ (*p* < 0.001) (Figure 1e).

Cell viability of adhered cells remained consistently high (>95%) across all conditions (B_1_, B_2_, and B_3_), with only slight fluctuations observed up to day 3, and no significant differences observed among experiments (*p* > 0.05) (Figure 1a–c). Viability was also assessed by AO/EB fluorescent staining. Accordingly, ASCs adhered to the MCs remained predominantly viable (green) at all evaluated time points: day 1 (Figure 1(g_1_–g_3_)), day 7 (Figure 1(g_4_–g_6_)), day 14 (Figure 1(g_7_–g_9_)), and day 21 (Figure 1(g_10_–g_12_)) across all experimental conditions. Apoptotic (orange) and necrotic (red) cells were infrequent but increased gradually with prolonged culture time (Figure 1(g_8_–g_10_,g_12_)), particularly in B_3_, which reached its highest cell density at 14 days and consequently showed a greater presence of apoptotic/necrotic cells within the dense microcarrier aggregates (Figure 1(g_9_)). As cell concentration and aggregate size decreased, the incidence of apoptotic or necrotic cells also decreased at day 21 in B_3_ (Figure 1(g_12_)). Viable cells exhibiting stained lysosomes were abundant (Figure 1(g_7_–g_12_)), although this feature was not observed in all analyzed cells.

Overall, ASC expansion in the bioreactor was primarily driven by the combined effects of oxygen availability and agitation rather than inoculum density alone. Among the evaluated conditions, B_3_ provided the most favorable environment, supporting higher growth rates, earlier peak productivity, and sustained cell expansion. These results identified B_3_ as the optimal condition for ASC cultivation and justify its selection for subsequent experiments.

### 2.2. Balanced Metabolism During the Early Exponential Growth Phase in B_3_ Supports Enhanced ASC Expansion Despite Progressive DNA Damage

**Figure 2:**
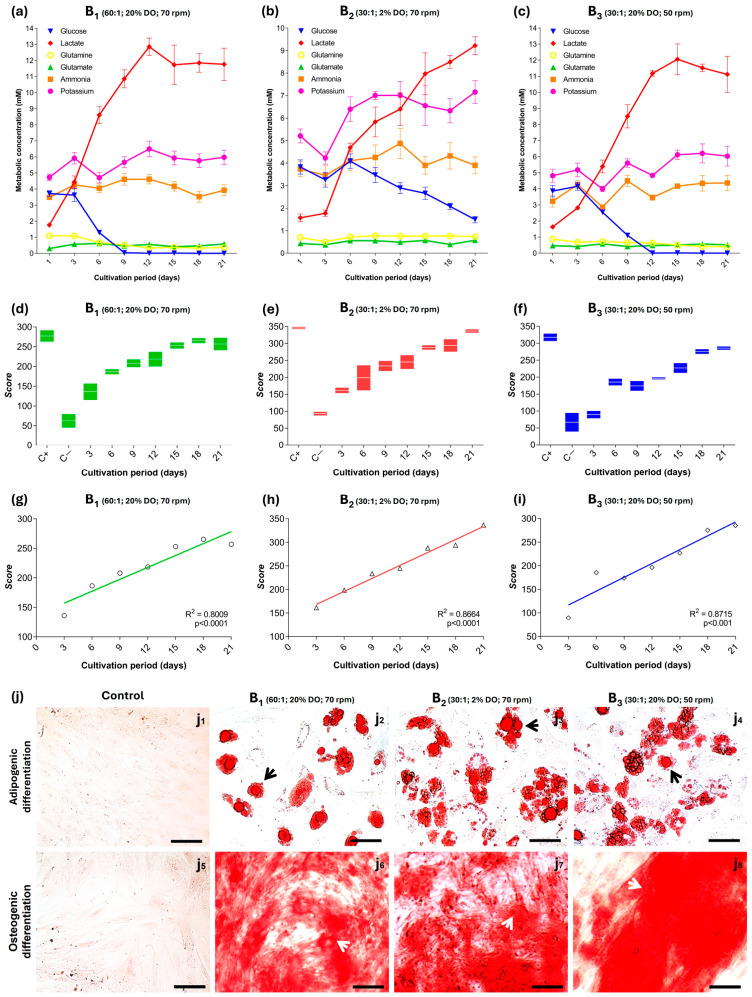
Metabolic profile, genotoxicity score, and differentiation potential of ASCs cultured under different bioreactor conditions. (**a**–**c**) Temporal metabolic profiles showing concentrations of glucose, lactate, glutamine, glutamate, ammonia, and potassium during the 21 days of cultivation under conditions B_1_ (60:1; 20% DO; 70 rpm) (**a**), B_2_ (30:1; 2% DO; 70 rpm) (**b**), and B_3_ (30:1; 20% DO; 50 rpm) (**c**). (**d**–**f**) DNA damage score progression over cultivation time for B_1_ (**d**), B_2_ (**e**), and B_3_ (**f**), including positive (C^+^) and negative (C^−^) controls. (**g**–**i**) Linear regression analysis correlating DNA damage score with cultivation time for each condition: B_1_ (**g**), B_2_ (**h**), and B_3_ (**i**), showing strong positive correlations (R^2^ values indicated in each graph). (**j**) Multilineage differentiation potential of ASCs after bioreactor cultivation. Adipogenic differentiation (**j_1_**–**j_4_**) was evidenced by lipid droplet accumulation stained in red, and osteogenic differentiation (**j_5_**–**j_8_**) was confirmed by mineralized matrix deposition. Control cells represent undifferentiated cells. Arrows indicate representative regions of differentiation. Scale bars: 50 µm (**j_2_**–**j_4_**,**j_8_**), 100 µm (**j_1_**,**j_5_**,**j_7_**), and 200 µm (**j_6_**).

The metabolic profiles observed in Figure 2a–c followed distinct patterns across the evaluated bioreactor conditions and were associated with the cell growth kinetics presented in Figure 1. In condition B_1_, a rapid decrease in glucose concentration was observed, accompanied by a marked accumulation of lactate over time (Figure 2a). This behavior was supported by the metabolic parameters obtained during the exponential phase, with high glucose consumption (Y_(Glu/X)_ = 5.11 pg/cell) coupled with elevated lactate production (Y_(Lac/X)_ = 6.92 pg/cell), while glutamine consumption remained negligible (Y_(Gln/X)_ = 0.00107 pg/cell) and ammonia generation was relatively low (Y_(Amn/X)_ = 0.15 pg/cell) (Table 2). This profile coincided with an initial increase in cell concentration, followed by a reduction in growth rate after glucose depletion, while cell viability remained high.

In condition B_2_, glucose consumption occurred more gradually, with continuous lactate production and greater accumulation of ammonia throughout the cultivation period (Figure 2b). This pattern was reflected in the phase-dependent metabolic parameters. During the first exponential phase, glucose consumption was low (Y_(Glu/X)_ = −1.26 pg/cell) with moderate lactate production (Y_(Lac/X)_ = 5.79 pg/cell) and ammonia generation (Y_(Amn/X)_ = 0.177 pg/cell). In the second phase, glucose consumption increased (Y_(Glu/X)_ = 7.17 pg/cell), while lactate production decreased (Y_(Lac/X)_ = 3.92 pg/cell) and ammonia accumulation became more pronounced (Y_(Amn/X)_ = 0.426 pg/cell). Glutamine utilization remained minimal in both phases (Y_(Gln/X)_ = −0.00066 and −0.00018 pg/cell), indicating limited dependence on this substrate (Table 2). This metabolic profile was accompanied by lower cell expansion and reduced final cell density compared with the other conditions.

Condition B_3_ showed a metabolic pattern characterized by rapid glucose consumption and elevated lactate accumulation, similar to B_1_, but with sustained cell growth over a longer period and higher final cell density (Figure 2c). This condition exhibited a more balanced metabolic profile across both exponential phases. In the first phase, moderate glucose consumption (Y_(Glu/X)_ = 2.99 pg/cell) was associated with lactate production (Y_(Lac/X)_ = 3.01 pg/cell) and negligible ammonia accumulation (Y_(Amn/X)_ = −0.076 pg/cell).

In the second phase, both glucose consumption and lactate production decreased (Y_(Glu/X)_ = 1.24 pg/cell; Y_(Lac/X)_ = 1.33 pg/cell), while ammonia levels remained low (Y_(Amn/X)_ = −0.051 pg/cell). Glutamine consumption was minimal in both phases (Y_(Gln/X)_ = 0.00005 and 0.00015 pg/cell), and glutamate variations were negligible throughout the cultivation period (Y_(Glm/X)_ ≈ 10^−4^ pg/cell). Together, these results indicate a more controlled metabolic state, supporting sustained cell expansion.

DNA damage in cells before and after cultivation in the bioreactor was evaluated using the comet assay, performed with the initial inoculum (negative control) and at days 3, 6, 9, 12, 15, 18, and 21 of culture for each experimental condition (B_1_, B_2_, and B_3_). As shown in Figure 2d, a significant time-dependent correlation (*p* < 0.001) was observed between the level of genetic material damage, and the progression of culture time in the bioreactor experiments. This increase in DNA damage was accompanied by progressive changes in the metabolic profile, including glucose depletion and accumulation of lactate and ammonia (Figure 2a–c), occurring in parallel with the exponential growth phase and subsequent culture progression under all evaluated conditions.

The in vitro differentiation potential of ASCs into adipogenic (Figure 2(j_2_–j_4_)) and osteogenic lineages (Figure 2(j_6_–j_8_)) was confirmed at the end of each experiment (B_1_, B_2_, and B_3_) following exposure to the respective induction media, as evidenced by lipid droplet accumulation and mineralized matrix deposition throughout the cytoplasm. Control cultures maintained under basal conditions did not exhibit morphological changes or staining patterns indicative of adipogenic or osteogenic differentiation (Figure 2(j_1_,j_5_)).

### 2.3. Optimized Short-Term Culture Enhances ASC Expansion and Metabolic Activity While Preserving Cellular Functionality Despite Increases in Genotoxic Markers

Based on the findings obtained from experiments B_1_ through B_3_, additional bioreactor cultures were conducted to optimize cell yield while preserving viability and minimizing DNA damage. Among the initial conditions, culture B_3_ provided the highest cell yield at 21 days; however, its pronounced proliferative capacity was accompanied by elevated levels of DNA fragmentation, as indicated by the comet assay, along with the formation of large MC aggregates that negatively affected the harvest of adherent cells.

Therefore, experiments B_4_ and B_5_ were designed with a shorter cultivation period (9 days), encompassing the first exponential growth phase observed under condition B_3_, which showed the best performance. In addition, to reduce aggregate formation and maintain cell viability, cultures were initiated at a lower cell density (1.2 × 10^4^ cells/mL, corresponding to approximately 15 cells per MC), while maintaining 20% DO throughout cultivation. The B_4_ and B_5_ conditions differed in their medium renewal strategies: in B_4_, medium replacement was performed, following the same protocol used for B_1_–B_3_ (25% every 3 days), whereas in B_5_, it was carried out every 2 days (25%) starting from day 3.

**Figure 3:**
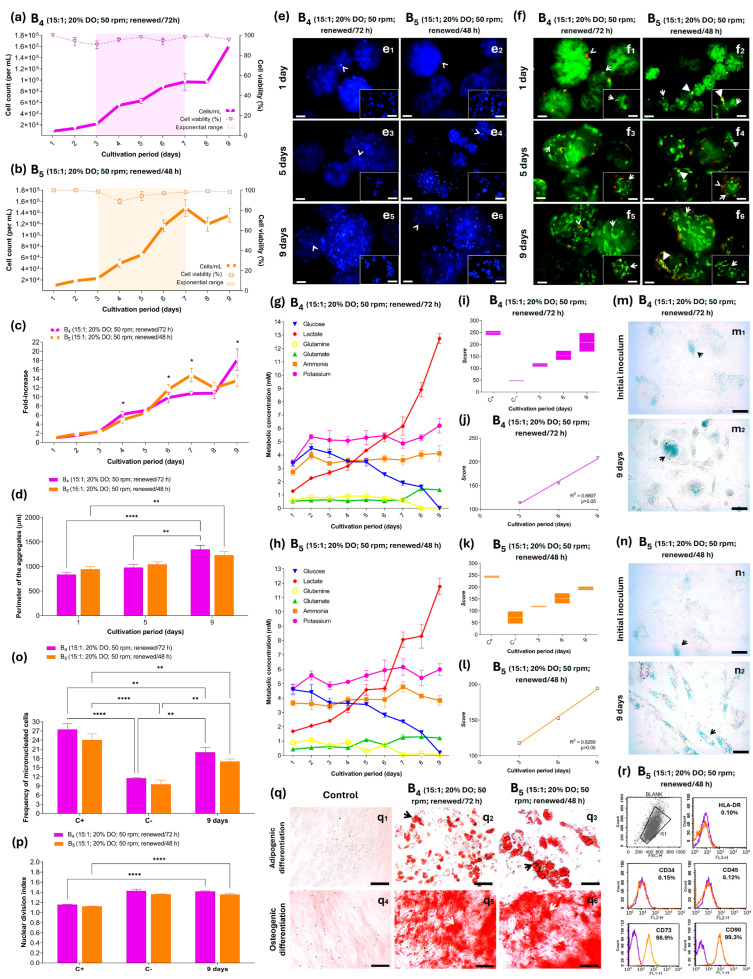
Optimization of initial inoculum and medium exchange strategy in ASC bioreactor cultivation: effects on growth, aggregation, metabolism, genomic integrity, senescence, differentiation potential, and immunophenotype. (**a**,**b**) Growth kinetics of ASCs cultured under condition B_4_ (15:1; 20% DO; 50 rpm; medium renewal every 72 h) (**a**) and B_5_ (15:1; 20% DO; 50 rpm; medium renewal every 48 h) (**b**), showing cell concentration (adherent viable cells/mL) and adherent cells viability (%) over 9 days of cultivation. Shaded areas indicate the exponential growth phase. (**c**) Fold increase relative to the extent to which the culture proliferated over time for B_4_ and B_5_ conditions. * Statistically significant difference (two-tailed *p* < 0.05). (**d**) Quantification of aggregate size, expressed as perimeter (µm), at different cultivation times (days 1, 5, and 9), showing progressive aggregate formation under both conditions. (**e**) Representative fluorescence microscopy images (DAPI staining, blue) illustrating cell distribution (thin arrowheads) and aggregate organization at 1, 5, and 9 days for B_4_ and B_5_. Insets highlight cellular density within aggregates. (**f**) AO/EB staining showing cell viability within MCs and aggregates over time. Viable cells fluoresce green (thin arrows), while apoptotic cells fluoresce yellow (thick arrowheads), and necrotic cells appear orange/red (thin arrowheads). Viable cells also showed lysosomes throughout the cytoplasm, colored in red (thin arrows). (**g**,**h**) Metabolic profiles during cultivation, showing concentrations of glucose, lactate, glutamine, glutamate, ammonia, and potassium for B_4_ (**g**) and B_5_ (**h**). (**i**–**l**) DNA damage assessment by comet assay, expressed as DNA damage scores over cultivation time for B_4_ (**i**,**j**) and B_5_ (**k**,**l**), including linear regression analysis. (**m**,**n**) Senescence-associated β-galactosidase assay showing senescent cells (black arrows) at the initial inoculum and after 9 days of cultivation for B_4_ (**m_1_**,**m_2_**) and B_5_ (**n_1_**,**n_2_**). Blue staining indicates β-galactosidase-positive (senescent) cells. (**o**) Frequency of micronucleated cells under positive (C^+^), negative (C^−^), and cultivation (9 days) conditions for B_4_ and B_5_. (**p**) Nuclear Division Index (NDI) for B_4_ and B_5_ conditions, indicating cell proliferation status. (**q**) Multilineage differentiation potential after bioreactor cultivation. Adipogenic differentiation (**q_2_**,**q_3_**) was evidenced by lipid droplet accumulation (red staining, black arrows), and osteogenic differentiation (**q_5_**,**q_6_**) was confirmed by mineralized matrix deposition (white arrows). Control represents undifferentiated cells (**q_1_**,**q_4_**). (**r**) Immunophenotypic characterization of B_5_ cells by flow cytometry, confirming expression of mesenchymal markers (CD73, CD90) and low expression of hematopoietic/immune markers (CD34, CD45, HLA-DR). For figures (**d**,**o**,**p**): *p* < 0.01 (**); and *p* < 0.0001 (****). Scale bars: 50 µm (**e_1_**–**e_3_**,**e_5_**,**e_6_**,**f_1_**,**f_3_**–**f_6_**,**m_1_**,**m_2_**,**n_1_**,**n_2_**,**q_1_**–**q_4_**), 80 µm (insets (**f_1_**–**f_6_**)), 100 µm (**e_4_**,**f_2_**,**q_5_**,**q_6_**), and 600 µm (insets (**e_1_**–**e_6_**)).

Figure 3 displays the growth profiles of cultures B_4_ and B_5_, along with cell viability throughout the 9-day cultivation period and the *FI*, including statistical comparisons between conditions. Table 2 summarizes the kinetic parameters of proliferation derived from the exponential growth phase of each culture.

Both cultures demonstrated comparable overall cell yields (Figure 3a,b); however, B_5_ reached its maximum cell productivity earlier (day 7) relative to B_4_ (day 9), resulting in a higher cell production rate in cells/mL/h (Table 2). Although both cultures exhibited an exponential proliferation phase over the same interval (Figure 3a,b), the population doubling time was shorter in B_5_ (36.5 h) compared with B_4_ (48.5 h) (Table 2). When comparing the *FI* (Figure 3c), B_4_ completed the 9-day cultivation period with a higher *FI* value than B_5_ (18.1 vs. 13.5; *p* > 0.05). Nonetheless, both B_4_ and B_5_ achieved an *FI* greater than those recorded in cultures B_1_, B_2_, and B_3_ at the same cultivation time (3.1, 3.9, and 7.8 at day 9, respectively). Regarding adherent cell viability, both cultures maintained values above 90% throughout the entire period (*p* > 0.05).

The density of ASCs adhered to the MCs was monitored at days 1, 5, and 9 under the B_4_ and B_5_ bioreactor conditions (Figure 3d,e). Due to the lower initial inoculum, a sparse distribution of adherent cells was observed on the MCs within the first 24 h of culture (Figure 3(e_1_,e_2_)). However, by day 5 (120 h), the MCs exhibited moderate cellular coverage, along with smaller MC aggregates (Figure 3d,(e_3_,e_4_)). By day 9, most MCs reached confluence levels above 70%, and larger aggregates (*p* < 0.01) were observed, interconnected by adhered cell populations bridging the macroporous structures (Figure 3d,(e_5_,e_6_)). No significant differences (*p* > 0.05) were observed between B_4_ and B_5_ in terms of MC aggregation over time and the mean aggregate perimeter at day 9 remained lower than that observed in the B_1_–B_3_ cultures at day 7 (>1500 μm) (Figure 1e and Figure 3d).

Cell viability of ASCs on MCs was monitored using AO/EB fluorescence staining (Figure 3f). A high incidence of viable adherent cells (green) was observed from the beginning up to day 9 f culture in both experimental conditions. Apoptotic cells (yellow) were sparse, though slightly more frequent in B_5_, without a time-dependent pattern (Figure 3(f_2_,f_4_,f_6_)). Necrotic cells (red) were rare, even within the larger MC aggregates (Figure 3(f_1_,f_3_–f_5_)). Notably, viable cells displaying an abundant presence of lysosomal vesicles intensely labeled in red were prominent throughout the culture period, particularly at day 9 (Figure 3(f_5_,f_6_)).

The metabolic behavior of ASCs under the modified cultivation strategies (B_4_ and B_5_) was also evaluated to assess the impact of shorter culture duration and different medium renewal regimes on nutrient consumption and byproduct formation (Figure 3g,h). In condition B_4_, glucose concentration decreased progressively throughout the cultivation period, accompanied by a moderate accumulation of lactate (Figure 3g). This behavior is consistent with the specific metabolic rates obtained during the exponential phase, with glucose consumption (Y_(Glu/X)_ = 3.86 pg/cell) and lactate production (Y_(Lac/X)_ = 4.18 pg/cell) indicating a moderately glycolytic metabolism. Glutamine consumption remained minimal (Y_(Gln/X)_ = 0.00057 pg/cell), and ammonia production was low (Y_(Amn/X)_ = 0.059 pg/cell), suggesting limited nitrogen metabolism and reduced accumulation of potentially toxic byproducts. The temporal metabolic profile, together with the controlled generation of lactate and ammonia, indicates a relatively stable metabolic state, supporting cell expansion under reduced metabolic stress.

In contrast, condition B_5_ exhibited a more pronounced metabolic activity, characterized by a faster decline in glucose concentration and a sharp increase in lactate levels over time (Figure 3h). Despite presenting a lower specific glucose consumption (Y_(Glu/X)_ = 1.89 pg/cell), cells showed higher lactate production (Y_(Lac/X)_ = 6.88 pg/cell), indicating a more glycolytic phenotype. Glutamine consumption remained negligible (Y_(Gln/X)_ = 0.00075 pg/cell), while ammonia production was markedly higher (Y_(Amn/X)_ = 0.331 pg/cell) compared with B_4_, reflecting increased metabolic turnover. This intensified metabolic profile is consistent with the higher cell productivity observed under this condition, although it may also suggest a greater accumulation of metabolic byproducts over time.

ASCs from the initial inoculum (negative control) and those recovered throughout culture (days 3, 6, and 9) under the B_4_ and B_5_ experimental conditions in the bioreactor were evaluated for DNA fragmentation using the comet assay. An increase in the DNA damage score of ASCs was observed as a function of culture time (Figure 3i–l), exceeding the values recorded for the negative control. By the end of culture (9 days), the scores approached those of the positive control, particularly in B_4_ (Figure 3i). When comparing the scores obtained for B_4_ and B_5_ at 9 days of culture with those from cultures B_1_ to B_3_ at the same time point, similar values were found, ranging between 200 and 250 (Figure 2d–f and Figure 3i,k). Despite the apparent time-dependent increase in DNA damage score observed graphically, the correlation test was not statistically significant (*p* > 0.05), likely due to the limited sample size.

The degree of senescence in ASCs before and after culture under the B_4_ and B_5_ bioreactor conditions was evaluated by staining for β-galactosidase activity, an enzyme typically associated with senescent cells. As shown in Figure 3m,n, β-galactosidase activity was scarce in the cells from the initial inoculum for both B_4_ and B_5_ (Figure 3(m_1_,n_1_)). However, after 9 days of culture, a greater proportion of ASCs recovered from the MCs exhibited positive staining, indicating increased β-galactosidase activity (Figure 3(m_2_,n_2_)). No qualitative differences were observed in the abundance of senescent cells between the B_4_ and B_5_ cultures.

Mutagenicity in ASCs cultured under the B_4_ and B_5_ bioreactor conditions was assessed using the micronucleus test. As shown in Figure 3o, the frequency of micronucleated cells increased (*p* < 0.01) after 9 days of culture compared with the initial inoculum (day 0; negative control, C^−^) in both experimental conditions (11.5 at day 0 vs. 20 at day 9 for B_4_, and 9.5 at day 0 vs. 17 at day 9 for B_5_). However, despite this increase in micronucleus frequency, the value was lower (*p* < 0.01) than that of the positive control (C^+^). No significant differences were detected between the B_4_ and B_5_ cultures at day 9 (*p* > 0.05).

Similar results were observed for the Nuclear Division Index (NDI). Both positive controls exhibited a lower (*p* < 0.0001) proportion of proliferating cells following exposure to the genotoxic agent Mitomycin C, when compared to the pre- and post-culture cells from B_4_ and B_5_, which did not differ from each other (*p* > 0.05) (Figure 3p). After 9 days of culture, the NDI in B_4_ was 1.41, compared with 1.42 in the pre-culture sample (C^−^), while in B_5_ the NDI was 1.36 for both the initial inoculum and for the cells recovered after 9 days of culture (Figure 3p).

The in vitro adipogenic and osteogenic differentiation potential of the ASCs recovered after bioreactor cultures B_4_ and B_5_ was confirmed by the specific staining of intracellular lipid droplets and calcium deposits, respectively (Figure 3q). In parallel, negative controls for each culture condition (cells maintained under the same protocol but without the differentiation medium) did not exhibit morphological changes or positive staining, confirming the absence of spontaneous differentiation (Figure 3(q_1_,q_4_)).

Finally, the immunophenotypic characterization of ASCs from the B_5_ bioreactor condition after 9 days of culture was performed by flow cytometry, and the results are shown in Figure 3r. The analyzed cells, derived from the same donor used in the previous experiments (B_1_ to B_4_), presented expression levels greater than 98% for the MSC-associated markers CD73 and CD90, and expression levels below 1% expression for HLA-DR, CD34, and CD45 (Figure 3r). These results are in accordance with the criteria established by the International Society for Cellular and Gene Therapy (ISCT) and demonstrate that large-scale bioreactor culture did not alter the cellular identity of the ASCs.

Although optimized conditions significantly improved ASC expansion and productivity, genotoxic analyses revealed a progressive accumulation of DNA damage over time. Notably, this increase occurred despite the maintenance of high viability, a stable immunophenotype, and preserved differentiation capacity, indicating that conventional quality metrics may not fully capture underlying genomic alterations arising during prolonged ASC expansion.

## 3. Discussion

Despite the increasing use of ASCs in cell therapy, current strategies for large-scale expansion remain primarily focused on maximizing cell yield while preserving classical phenotypic attributes. In this context, our previous work established small-scale suspension culture conditions that improved ASC expansion while maintaining identity and differentiation capacity [47]. Building on this foundation, the present study advances toward scalable bioreactor cultivation by systematically optimizing key operational parameters. Importantly, this work integrates genotoxicity assessment into the bioprocess evaluation, enabling a comprehensive analysis of cell expansion performance alongside genomic integrity, thereby addressing a critical gap in current bioprocessing approaches.

In line with the optimization of bioreactor parameters described above, the initial cell-to-microcarrier ratio was evaluated as a critical factor influencing ASC expansion. Cell adhesion to MCs is a stochastic process that follows a Poisson distribution, resulting in a heterogeneous distribution of cells per carrier [48]. To reduce the proportion of empty MCs, higher cell-to-bead ratios are often used; however, excessively high ratios increase the number of unattached cells, leading to cell loss due to subsequent death [49]. Therefore, identifying a ratio that balances MC colonization and minimizes cell wastage is essential. In the bioreactor experiments, an initial ratio of 60:1 (B_1_) was tested, followed by lower ratios of 30:1 (B_2_ and B_3_) and 15:1 (B_4_ and B_5_). Consistent with previous small-scale findings in spinner flasks [47], the 60:1 condition resulted in lower adhesion efficiency and poor performance in the bioreactor, reaching a maximal *FI* of only 5.3. In contrast, the 30:1 and 15:1 ratios supported significantly higher cell expansion, with *FI* values of 18.4 (B_3_) and 18.1 (B_4_), respectively. The reduced inoculum also effectively limited aggregate size, which is critical for maintaining cell viability and improving productivity. At 9 days of culture, aggregate dimensions in B_4_ and B_5_ were considerably smaller than those observed in B_1_–B_3_ at day 6, despite B_1_–B_3_ beginning with a higher inoculum and showing lower cell concentrations during comparable early culture periods. Based on these results, the 15:1 cell-to-MC ratio was selected as the optimal condition for bioreactor cultures.

Agitation plays a key role in establishing a homogeneous physicochemical environment in culture systems by enhancing mass transfer and reducing concentration gradients [50]. It also increases the exposure of the MC surface to cells, favoring adhesion, while moderate agitation promotes convective flow through macroporous MCs, facilitating oxygen and nutrient transport as well as waste removal [49]. However, excessive agitation (>100 rpm) can generate high shear forces, leading to cell detachment and increased debris formation. Based on these considerations, two agitation conditions (50 and 70 rpm), both sufficient to maintain full MC suspension, were evaluated. Initial bioreactor experiments conducted at 70 rpm (B_1_ and B_2_) were followed by a reduction to 50 rpm combined with a lower inoculum ratio (30:1), resulting in a marked increase in cell yield in B_3_. Therefore, 50 rpm was selected as the standard agitation speed for subsequent experiments.

Following the definition of physical parameters, DO concentration was investigated due to its central role in cellular metabolism and expansion [51]. Although hypoxic conditions (2–5% DO) have been reported to enhance ASC proliferation, differentiation, genomic stability, and anti-inflammatory secretome under hypoxic conditions in static cultures [52,53,54,55,56,57], other studies indicate that hypoxia may impair proliferation and downregulate genes involved in DNA repair and damage response pathways [52,58,59].

In this study, the initial condition (B_1_) was performed under normoxia (20% DO), followed by a hypoxic condition (2% DO) in B_2_. Under hypoxia, ASC expansion was initially accelerated, reaching a doubling time of 51.3 h during the first exponential phase. This was accompanied by higher extracellular potassium levels compared with the other conditions, particularly during the exponential growth phase, suggesting alterations in potassium flux associated with cellular adaptation to low oxygen levels. This observation is consistent with previous reports showing that hypoxia modulates outward potassium currents in ASCs, affecting their electrophysiological behavior and potentially influencing proliferation dynamics [60]. However, in B_2_, the initial exponential phase was followed by a marked decline in proliferation, despite the availability of nutrients and low accumulation of inhibitory metabolites. This behavior likely reflects oxygen diffusion limitations within MC aggregates, leading to localized severe hypoxia and reduced proliferative capacity. As a result, B_2_ exhibited low productivity with a maximum *FI* of 6.7. When DO was restored to 20% in B_3_, using the same initial inoculum as B_2_, cell expansion improved substantially, although a sharp decrease in cell concentration occurred after 14 days, likely due to nutrient depletion and metabolic accumulation associated with an insufficient feeding strategy. Overall, these results indicate that while hypoxia may enhance early ASC proliferation, normoxic conditions are required to sustain cell expansion and achieve higher productivity in MC-based bioreactor cultures.

Defining an appropriate medium-exchange regimen is essential to maintain adequate nutrient availability and prevent the accumulation of inhibitory metabolites during MSC proliferation [61]. Key substrates such as glucose and glutamine must be maintained above limiting concentrations, whereas lactate and ammonia—byproducts known to impair mammalian cell growth—should be minimized [49]. Our previous studies demonstrated that both static and suspension cultures can achieve similar yields even under conditions of nutrient excess [47,62], highlighting the importance of balancing nutrient replenishment with the removal of metabolite-rich medium to reduce costs and avoid unnecessary medium consumption. In the bioreactor experiments, cultures B_1_–B_4_ employed a 25% medium renewal every 72 h. Under this regimen, a progressive decrease in glucose levels and accumulation of lactate were observed during the exponential growth phase, indicating a predominantly glycolytic metabolism, while maintaining high cell viability and cell yield.

To evaluate potential improvements in productivity within the 9-day cultivation period, condition B_5_ was operated with 25% medium renewal every 48 h. Comparison between B_4_ and B_5_ revealed distinct growth patterns: although B_4_ reached a slightly higher final cell concentration at day 9, B_5_ showed superior performance during the exponential phase, characterized by a shorter doubling time (36.3 h vs. 48.5 h), higher μ_max_, and earlier attainment of maximum productivity. Based on these parameters, B_5_ was defined as the optimized culture strategy.

The improved performance observed in B_5_ is likely associated with the timing of nutrient replenishment, which occurred at days 3 and 5, corresponding to the early and mid-exponential phases. In contrast, B_4_ received medium renewal at the beginning and near the end of the exponential phase (day 6). However, previous studies have reported a limited influence of medium-exchange frequency on final cell density. For instance, dos Santos et al. [22] observed similar cell concentrations in cultures subjected to daily renewal, renewal every 48 h, or supplemented with nutrient concentrates, suggesting that longer intervals between medium exchanges may favor the accumulation of autocrine factors that support MSC proliferation.

Equally important as defining critical process parameters is ensuring that key quality attributes are met to guarantee the safety and efficacy of cell-based products. However, only a limited number of studies have addressed quality attributes related to the genetic stability of MSCs, such as karyotypic integrity or telomerase activity [53,61]. A major challenge in MSC-based therapies is the expansion of sufficient numbers of high-quality cells, particularly in the context of donor age–related decline, culture-induced senescence, or a combination of both [33]. Cellular senescence increases with successive divisions and can be further accelerated by intrinsic factors, including metabolic conditions and reactive oxygen species (ROS) generation, as well as extrinsic culture parameters such as pH, temperature, oxygen availability, cell adhesion, and mechanical forces [63].

Despite the recognized sensitivity of genotoxicity assays such as the comet assay, which reflects DNA damage associated with senescence [40], and the micronucleus test, which detects mutagenic events [36], these approaches remain underexplored in the context of MSC bioprocess optimization. Consequently, the relationship between expansion performance and genomic integrity during large-scale culture remains insufficiently characterized.

In the present study, DNA fragmentation assessed by the comet assay increased in a time-dependent manner, with prolonged culture consistently associated with higher levels of genetic damage, regardless of cultivation parameters. This pattern is consistent with previous observations in senescent ASCs [39,41] and other MSC sources [40,64]. The progressive increase in DNA damage observed during culture may be associated with multiple interacting factors. Increasing cell density and aggregate formation may create localized gradients of oxygen and nutrients, promoting metabolic stress and the accumulation of byproducts. These conditions may contribute to increased ROS generation, which has been implicated in DNA damage accumulation and senescence processes. Additionally, prolonged exposure to hydrodynamic forces generated during stirred bioreactor cultivation may represent an additional source of cellular stress. Although these mechanisms were not directly evaluated in the present study, they represent plausible contributors to the progressive genomic alterations observed during ASC expansion. Notably, this progressive accumulation of DNA damage occurred alongside sustained cell proliferation and preserved phenotypic characteristics, reinforcing the notion that conventional expansion metrics may not necessarily reflect underlying genomic alterations.

In parallel, senescence-associated β-galactosidase staining revealed an increase in senescent cells at later time points, supporting the association between prolonged culture, metabolic stress, and the onset of cellular senescence. The accumulation of SA-β-Gal–positive ASCs with increasing passage number has been previously reported [38], and its association with increased lysosomal content [65] is consistent with the time-dependent accumulation of lysosomes observed in our AO/EB staining. Collectively, these findings support the progressive establishment of a senescence-associated phenotype during in vitro expansion and highlight that the incorporation of complementary assays, such as p16 and p21 expression, ROS quantification, telomerase activity, or additional DNA damage markers, should be considered in future investigations to further refine senescence assessment in bioprocess settings.

Additionally, the micronucleus (MN) assay revealed an increase in chromosomal damage following bioreactor cultivation (B_4_ and B_5_), although values remained significantly lower than those observed in the positive control. These findings are consistent with previous reports describing MN formation in long-term ASC cultures [38]. Together with the comet assay results, these data indicate that genomic alterations during ASC expansion extend beyond DNA strand breaks to include mutagenic events at the chromosomal level. In this context, prolonged culture appears to be associated with cumulative metabolic and proliferative stress, contributing to the progressive increase in senescence and genomic instability observed in ASC populations. Future studies integrating molecular profiling with bioprocess optimization may help elucidate the mechanisms associated with DNA damage accumulation during prolonged ASC expansion.

Despite the progressive accumulation of DNA damage and micronucleus formation, flow cytometry and differentiation assays confirmed that ASCs maintained their mesenchymal identity following bioreactor expansion according to ISCT criteria [1]. These results reinforce that conventional quality attributes remain preserved even in the presence of detectable genomic alterations, although their long-term biological consequences remain to be fully elucidated. Therefore, the detection of genotoxic and senescence-associated alterations highlights the importance of carefully defining culture duration and process conditions to ensure the maintenance of cell quality for downstream applications.

## 4. Materials and Methods

### 4.1. Collection and Processing of Adipose Tissue

ASCs were isolated from adipose tissue as previously described [47,62]. Samples were obtained from healthy female donors who underwent elective abdominal dermolipectomy at an aesthetic surgery clinic located in Assis, São Paulo, Brazil. All participants received detailed information about the study and provided written informed consent authorizing the use of their biological material. The collected adipose tissue was sectioned into smaller fragments and transferred to phosphate-buffered saline (PBS; pH 7.2; 13-30258-05, LGC Biotecnologia, Cotia, Brazil) supplemented with 2% antibiotic-antimycotic solution (AA; BR30331-01, LGC Biotecnologia, Cotia, Brazil). Samples were maintained at 4 °C for 2 h to minimize the risk of microbial contamination.

### 4.2. Isolation and Culture of ASCs

The minced adipose tissue was digested enzymatically using a 0.15% collagenase type I solution (17018-029, Gibco, New York, NY, USA) and incubated in a thermostatic water bath at 37 °C for 1 h and 30 min. Following digestion, the material was centrifuged at 676× *g* for 5 min, after which the supernatant was discarded. The resulting pellet was resuspended in α-modified Eagle’s medium (αMEM; BR30007-05, LGC Biotecnologia, Cotia, Brazil) supplemented with 10% fetal bovine serum (FBS; 10Bio500, LGC Biotecnologia, Cotia, Brazil) and 1% AA. The suspension was subsequently passed through a 70 µm cell strainer (352350, BD Falcon, Mississauga, ON, Canada) to remove residual debris and centrifuged again at 314× *g* for 5 min.

The cell pellet obtained was resuspended in complete αMEM medium containing 10% FBS and 1% AA and seeded into T-flasks (150–182 cm^2^). Cultures were maintained at 36.5 °C in a humidified atmosphere of 5% CO_2_ for 24 h to allow cell adhesion. After this initial period, non-adherent cells and cellular debris were removed by washing the adherent primary ASCs with PBS containing 1% AA for 10 min. The PBS was then replaced with fresh basal αMEM medium supplemented with 10% FBS and 1% AA. Cultures were maintained under the same incubation conditions, with partial medium renewal (50%) every 48 h to support cell proliferation.

### 4.3. Trypsinization, Cryopreservation, and Thawing of ASCs

When cultures reached approximately 80–90% confluence in T-flasks, adherent cells were rinsed with PBS and detached using a 0.25% trypsin–EDTA solution (T4049-500ML; Sigma-Aldrich, Darmstadt, Germany). Enzymatic activity was neutralized by adding αMEM basal medium supplemented with 10% FBS and 1% AA. The resulting cell suspension was centrifuged at 314× *g* for 5 min, and the obtained pellet was resuspended in fresh culture medium. Cell viability and density were determined using a Neubauer chamber and the Trypan Blue exclusion assay. Cells from the first passage were then cryopreserved in a freezing solution composed of 70% αMEM, 20% FBS, and 10% sterile dimethyl sulfoxide (DMSO), and stored in liquid nitrogen at −196 °C.

For subsequent experimental procedures, cryopreserved vials were thawed at room temperature. The thawed suspension was centrifuged at 392× *g* for 5 min, and the pellet was resuspended in PBS to remove residual DMSO. After a second centrifugation under the same conditions, the cell pellet was resuspended in basal αMEM medium supplemented with 10% FBS and 1% AA. The ASCs (passage 1) were then seeded into T-flasks and expanded until reaching the cell density required for inoculation into bioreactors for suspension culture experiments.

### 4.4. Recovery and Quantification of Cells in Static and Suspension Cultures

Cell quantification in both static and suspension culture systems was performed using a Neubauer chamber as we previously described [47,62]. For static cultures maintained in T-flasks, samples were obtained following cell detachment by trypsinization. Ten microliters of the resulting cell suspension in culture medium were collected for counting.

In suspension cultures maintained in bioreactors, daily sampling was performed by collecting 2 mL of culture medium under continuous agitation. The sample was then passed through a 70 µm cell strainer to separate cells adhered to the Cultispher-S^®^ microcarriers (DG-2001-OO, Percell Biolytica, Åstorp, Sweden) from non-adherent cells that passed through the filter. The retained MCs were transferred to a centrifuge tube by inverting the filter and washing with PBS. After sedimentation and removal of the PBS, 1 mL of 0.25% trypsin–EDTA solution was added to the tube, and the mixture was incubated in a thermostatic bath at 37 °C until the MCs were fully digested (15–30 min, with occasional manual agitation). The enzymatic activity was neutralized, followed by centrifugation at 392× *g* for 5 min. The resulting pellet was resuspended in 1 mL of basal medium, of which 10 µL was taken for counting to determine the viability of the cells adhered to the MCs.

For both culture conditions, 10 µL of the cell suspension was mixed with 10 µL of trypan blue solution (0.4%) to exclude non-viable (blue-stained) cells. Subsequently, 10 µL of the stained mixture was loaded into a Neubauer chamber, and viable cells were quantified across five quadrants using an optical light microscope [66].

The concentration of viable cells per milliliter in static cultures was calculated using Equation (1), while the total number of viable cells in the culture was obtained by multiplying this concentration by the total volume of the post-trypsinization cell suspension. In bioreactors, the concentration of viable cells per milliliter—both adherent and non-adherent—was determined using Equation (2), and the total number of viable cells was calculated according to Equation (3).

(1) $$X=\frac{N}{Q}\times Df\times 10^{4}$$

(2) $$X=\frac{N}{Q}\times Df\times 10^{4}÷2$$

(3) Xc=X×V

where *X* represents the concentration of viable ASCs (cells/mL) in either static or suspension culture; *N* corresponds to the total number of viable cells counted across the five quadrants of the Neubauer chamber; *Q* denotes the number of quadrants analyzed (*Q* = 5); *Df* is the dilution factor applied to the cell suspension counted with trypan blue (10 µL of sample mixed with 10 µL of trypan blue, resulting in a *Df* of 2); 10^4^ is the Neubauer chamber conversion factor (mL^−1^); and 2 accounts for the division by the sampling volume collected under agitation, yielding the final viable cell concentration (*X*) per milliliter. *Xc* indicates the total number of viable ASCs in the suspension culture, and *V* represents the total volume of culture medium in the bioreactor, multiplied by the viable cell concentration (*X*) obtained from Equation (2).

Cell viability (*Cv*, expressed as a percentage) was determined for each quantification by calculating the ratio between the number of viable cells (*Vc*) and the total number of cells (*Tc*)—including both viable and non-viable populations—multiplied by 100, as described in Equation (4):

(4) $$Cv=\frac{Vc}{Tc}\times 100$$

Cell counts were performed in quadruplicate for each sample. Based on these equations, growth curves were generated to illustrate the variation in viable cell concentration (expressed as cells/mL) over the cultivation period for each experimental condition, representing the proliferation dynamics of ASCs in suspension culture. In addition, the corresponding cell viability (%) was determined for each time point.

### 4.5. Microcarriers

Cultispher-S^®^ cross-linked macroporous gelatin-based microcarriers (MCs) were used in the bioreactor suspension culture experiments. The technical specifications of this product have been previously described [47]. MCs were used at a concentration of 1 g/L, corresponding to a surface area of 15,000 cm^2^/g of culture medium. Before experimental use, MCs were prepared according to the manufacturer’s protocol with minor modifications. The MCs were hydrated with distilled water (50 mL/g) for 1 h and sterilized in an autoclave for 20 min at 120 °C and 14.2 psi. Then, the MCs were washed twice with PBS, followed by conditioning with basal medium (αMEM) supplemented with 10% FBS and 1% AA for 2 h in a thermostatic bath at 37 °C.

### 4.6. Cell Adhesion Analysis

To evaluate ASC attachment to the MCs in bioreactor suspension culture, the fluorescent DNA-binding dye DAPI (4′,6-diamidino-2-phenylindole dihydrochloride; 300 nM; D1306, Invitrogen, Carlsbad, CA, USA) was employed. Briefly, 1 mL of culture medium was collected under gentle agitation, and after sedimentation of the MCs, the supernatant was removed. Subsequently, 300 μL of DAPI solution was added to the pellet and incubated for 10 min. The staining solution was then discarded, and the MCs were carefully washed twice with PBS to remove residual dye.

Images were acquired using a Zeiss Scope A1-Axio microscope (Zeiss, Oberkochen, Germany) equipped with an AxioCam ICc3 (3.0-megapixel) digital camera and the Axio Vision software, version 4.7.2 (Zeiss, Oberkochen, Germany). Observations were made using an ultraviolet filter set with DAPI excitation at 372 nm and emission at 456 nm.

### 4.7. Measurement Analysis of Microcarrier Aggregates

From the samples collected at 1, 7, 14, and 21 days for cultures B_1_–B_3_, and at 1, 5, and 9 days for cultures B_4_ and B_5_—originally prepared for fluorescent staining to assess cell density (previous section) and viability of adherent cells (Section 4.4)—the perimeter (µm) of the aggregated MCs was also measured. For each sample, between 20 and 50 Cultispher-S^®^ microcarrier aggregates formed by adherent ASCs were analyzed using the ImageJ software version 1.54p (National Institutes of Health, Bethesda, MD, USA), based on fluorescence micrographs captured with the digital imaging system described previously.

### 4.8. Culture of ASCs in Bioreactor

To evaluate the large-scale yield of ASC cultures under suspension conditions, a mechanically stirred and aerated tank-type bioreactor with a 1 L working capacity (New Brunswick BioFlo/CelliGen 115—Eppendorf, Hamburg, Germany) was employed. The system was equipped with a water-jacketed temperature control unit and a centrally fixed impeller with three blades set at a 45° angle. Prior to use, the bioreactor was assembled and sterilized in an autoclave for 25 min at 120 °C and 14.2 psi, then allowed to equilibrate overnight at room temperature to cool the water jacket and vessel interior before inoculation.

The bioreactor setup included a medium removal line extending to the bottom of the vessel, a sampling line positioned at the 300 mL level, and an inoculation line located at the headplate. These lines were connected via sterilized silicone tubing to peristaltic pumps with controlled rotation. The system also featured sensors for monitoring liquid temperature and gas flow, including an inlet for compressed air, CO_2_, N_2_, and O_2_—filtered through a 0.22 µm membrane—and an exhaust line equipped with a moisture condenser. Sensors for pH and dissolved oxygen (DO, expressed as percentage of air saturation) were calibrated prior to cell inoculation.

For DO calibration, the 100% set point was established after signal stabilization with a 600 mL working volume containing the MCs, constant agitation at 50 or 70 rpm (depending on the experiment), and 100% air injection. The zero set point (0%) was defined electronically by disconnecting the sensor cable until the reading stabilized at 0.0. Temperature, gas composition, pH, DO, and agitation parameters were controlled and monitored via the electronic interface of the bioreactor’s control cabinet.

This interface also allowed continuous monitoring and recording of all parameters throughout the culture. The data exported in spreadsheet format were analyzed to determine the mean value of each parameter at the end of cultivation and to compare them with the established set points (Table 1), allowing the correlation of variations in operating conditions and cell yield.

pH regulation was achieved through automatic CO_2_ injection, triggered when the pH exceeded the set point (7.3 ± 0.02). The use of a basic buffer solution was discontinued after it was observed that, in the absence of CO_2_, the culture medium naturally tended to alkalinize. The level sensor was also deactivated, as gas dispersion through the vessel headplate—rather than through a submerged sparger—prevented bubble formation, maintained efficient pH and DO control, and minimized shear stress.

Table 1 summarizes the fixed operational parameters applied to all bioreactor cultures, as well as the variable parameters specific to each experimental run (B_1_–B_5_). ASCs used in this study were obtained from a previously established cell bank generated from six independent donors. Each experiment was performed independently, as sequential single-run optimization experiments according to cell availability (2nd or 3rd passage), with protocol adjustments made progressively based on previous results and literature reports [47,62] to optimize the large-scale ASC cultivation process. Therefore, culture conditions were not designed to assess donor-specific biological variability. Technical replicates varied according to the assay performed and are specified in the corresponding methodological sections.

Based on our previous observations in spinner flask cultures [47], all cultures followed an intermittent adhesion regime lasting 6 h; however, the total working volume (600 mL) was present from the beginning of the process due to prior DO sensor calibration. Dissolved oxygen levels were selected to compare normoxic (20% DO) and hypoxic (2% DO) conditions due to their known effects on MSC proliferation and biological behavior. Similarly, different cell-to-microcarrier inoculum ratios were evaluated based on their expected impact on cell attachment efficiency, aggregate formation, and expansion kinetics.

### 4.9. Kinetic Analysis of Cell Proliferation

The proliferative behavior of ASCs in suspension culture was assessed through kinetic analysis by calculating the specific growth rate (*µₓ*) using Equation (5). In this equation, *X* corresponds to the cell concentration determined by counting in a Neubauer chamber, and *dlnX*/*dt* represents the rate of cell proliferation within the culture system.

(5) $${µ}_{x}=\frac{dlnX}{dt}$$

The maximum specific growth rate (*µ_max_*) was determined during the exponential (logarithmic) growth phase, where the growth rate remains constant. This was calculated by performing a linear regression of the natural logarithm of cell concentration (lnX) against cultivation time [67]. The slope of this linear fit, obtained from the plot of *ln*(*X*/*X*_0_) versus time, corresponds to *µ_max_*. This coefficient was also used to calculate the cell doubling time (*Dt*), or generation time, according to Equation (6).

(6) $$Dt=\frac{ln\;2}{{µ}_{max}}$$

The maximum cell productivity (*Π*), as defined in Equation (7), represents the rate of cell production from the initial cell concentration (*X_i_*) to the maximum cell concentration (*X_max_*) over the cultivation period required to reach this value (Δ*t_i_* → *t_max_*).

(7) $$\Pi =\frac{X_{max}-X_{i}}{{∆t}_{t_{i}\to t_{max}}}$$

The fold-increase (*FI*) reflects the extent of culture proliferation by comparing the number of cells at a given time (*Nf*) to the number of cells initially adhered after 24 h of culture (*Ni*). *FI* can be calculated daily using either the concentration of viable cells per milliliter or the total number of viable cells, as described in Equation (8).

(8) $$FI=\frac{Nf}{Ni}$$

### 4.10. Biochemical Analysis of Nutrients and Metabolites

The concentrations of key nutrients and metabolites were measured throughout the bioreactor cultures in triplicate. Samples were collected immediately prior to medium renewal according to each feeding regime. For B_1_–B_3_ conditions, sampling was performed on days 1, 3, 6, 9, 12, 15, 18, and 21, whereas for B_4_ and B_5_ conditions, samples were collected daily from day 1 to day 9. Briefly, 1 mL of culture medium was collected after halting agitation and allowing the MCs to settle. Samples were centrifuged at 750× *g* for 5 min, and the supernatant was filtered through a 0.22 µm membrane and stored at −20 °C until analysis.

Glucose, lactate, glutamine, glutamate, ammonia, and potassium concentrations (mM) were determined using a biochemical analyzer based on enzymatic reactions coupled with electrochemical detection (YSI 2950D Biochemistry Analyzer, YSI Life Sciences, Yellow Springs, OH, USA) [68]. Potassium concentration was monitored as an indicator of ionic balance and cell membrane integrity rather than as a metabolic substrate and therefore was not used to calculate yield coefficients.

Glucose and glutamine consumption, as described in Equation (9), as well as lactate, glutamate, and ammonium production, as described in Equation (10), were estimated based on the variation in metabolite concentrations between sampling points during the exponential growth phase and normalized to cell growth over the same period. Due to the fed-batch operation mode, with periodic partial medium replacement, the calculated values represent apparent specific consumption or production rates under dynamic culture conditions.

(9) $$Y_{\frac{N}{x}}=\frac{∆N}{{∆X}_{v}}=\frac{N_{{(t}_{1})}-N_{{(t}_{2})}}{{X_{v}}_{{(t}_{2})}-{X_{v}}_{{(t}_{1})}}$$

(10) $$Y_{\frac{M}{x}}=\frac{∆M}{{∆X}_{v}}=\frac{M_{{(t}_{2})}-M_{{(t}_{1})}}{{X_{v}}_{{(t}_{2})}-{X_{v}}_{{(t}_{1})}}$$

where *N*(*t*) is the nutrient concentration, *M*(*t*) is the metabolite concentration, and *Xv*(*t*) is the viable cell yield at a given time point during the beginning (*t*_1_) and end (*t*_2_) of the exponential growth phase [68].

### 4.11. Fluorescent Staining Analysis for Cell Viability on Microcarriers

**Figure 4:**
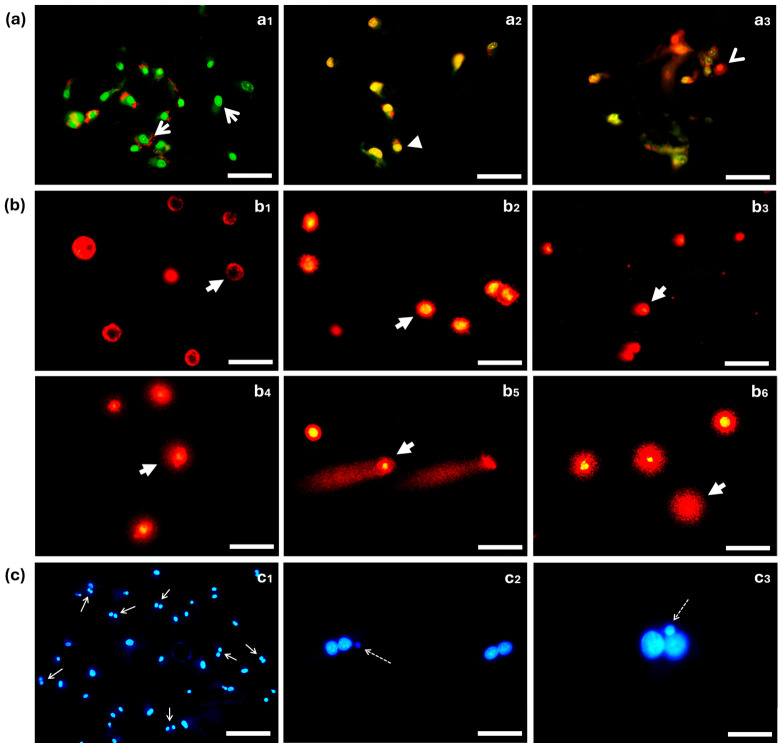
Criteria for assessment of cell viability, DNA damage, and genomic instability in ASCs cultured on MCs in the bioreactor. (**a**) Cell viability evaluated by Acridine Orange/Ethidium Bromide (AO/EB) double staining. Viable cells (thin arrow) exhibit green fluorescence (**a_1_**), apoptotic cells (thick arrowhead) show yellow/orange fluorescence (**a_2_**), and necrotic cells (thin arrowhead) fluoresce red (**a_3_**). The staining also highlights lysosomal structures in red (thin arrow). (**b**) DNA damage analysis by comet assay, qualitatively classified according to tail extent (thick arrows): Class 0 (no damage) (**b_1_**), Class 1 (low damage) (**b_2_**), Class 2 (intermediate damage) (**b_3_**), Class 3 (damage area approximately twice the head diameter) (**b_4_**), and Class 4 (no visible head or damage/tail area ≥ three times the head diameter) (**b_5_**,**b_6_**). (**c**) Genomic instability assessment by the micronucleus assay. (**c_1_**) Binucleated cells (thin long arrow) marked with the DNA-binding dye DAPI (blue color) were observed under fluorescence microscopy. (**c_2_**,**c_3_**) Representative micronuclei (dotted arrows) in binucleated cells. Scale bars: 10 µm (**c_2_**,**c_3_**), 50 µm (**a_1_**–**a_3_**,**b_1_**–**b_6_**,**c_1_**).

Cell viability of ASCs on microcarriers during bioreactor cultivation was monitored using a fluorescent staining method with Acridine Orange (3,6-dimethylaminoacridine) combined with Ethidium Bromide (3,8-diamino-5-ethyl-6-phenylphenanthridinium) (AO/EB). This dye combination allows the visualization of cells at different viability stages: viable cells fluoresce green (Figure 4(a_1_)), apoptotic cells fluoresce orange (Figure 4(a_2_)), and necrotic cells fluoresce red (Figure 4(a_3_)). Additionally, the staining labels lysosomal structures in red (Figure 4a).

The technique was applied at 1, 7, 14, and 21 days for cultures B_1_–B_3_ and at 1, 5, and 9 days for cultures B_4_–B_5_. For staining, 1 mL of culture medium was sampled under agitation, and after settling of the MCs, the medium was removed, and the MCs were washed once with PBS. After a second decantation and PBS removal, 300 µL of AO/EB solution (50 µg/mL) was added for 5 min. The staining solution was then discarded, and the MCs were gently washed twice with PBS. Visualization was performed using a fluorescence microscope with a blue filter for acridine orange (excitation: 490 nm; emission: 530 and 640 nm) and a green filter for ethidium bromide (excitation: 545 nm; emission: 605 nm). Images obtained for both dyes were merged using ImageJ software version 1.54p.

### 4.12. Comet Assay Analysis

To monitor in vitro DNA fragmentation of ASCs during bioreactor cultivation, the alkaline comet assay was performed following the protocol described by Fuchs and colleagues [40]. Briefly, 100 µL of the cell suspension obtained from MC digestion in 2 mL of culture medium sampled under agitation was mixed with 75 µL of 0.5% low-melting-point (LMP) agarose at 37 °C and spread onto pre-gelatinized slides coated with normal-melting-point (NMP) agarose. Two slides were prepared per sample. Samples were collected every 3 days of culture—at 3, 6, 9, 12, 15, 18, and 21 days for experiments B_1_–B_3_ and up to 9 days for cultures B_4_ and B_5_.

Slides were covered with coverslips and allowed to solidify at 4 °C for 15 min. Subsequently, slides were subjected to a lysis solution at 4 °C for 2 h, followed by immersion in NaOH buffer (pH > 13) for 20 min. Electrophoresis was performed at 20 V and 300 mA for 30 min. After electrophoresis, slides were neutralized with 0.4 M Tris buffer for 10 min, fixed in absolute ethanol for 10 min, and stained with 0.02 M ethidium bromide. A total of 200 nucleoids were randomly analyzed (100 per slide) using a fluorescence microscope at 40× magnification with a green filter to detect ethidium bromide fluorescence (excitation: 545 nm; emission: 605 nm).

DNA damage was qualitatively classified based on the extent of the damaged area: Class 0 (undamaged cells) (Figure 4(b_1_)), Class 1 (slightly damaged) (Figure 4(b_2_)), Class 2 (intermediate damage) (Figure 4(b_3_)), Class 3 (damage area approximately twice the diameter of the comet head) (Figure 4(b_4_)), and Class 4 (head not visible or a damage/tailed area ≥ three times the head diameter) (Figure 4(b_5_,b_6_)).

An arbitrary unit score (0 = no damage, 400 = maximum damage) was calculated for each slide to determine the DNA damage index (score), according to Equation (11).

(11) $$AU=\left(N0\times 0\right)+\left(N1\times 1\right)+\left(N2\times 2\right)+\left(N3\times 3\right)+(N4\times 4)$$

where *AU* (arbitrary units) represents the DNA damage score of the sample, and *N*_0_ to *N*_4_ correspond to the number of nucleoids in Classes 0, 1, 2, 3, and 4, respectively.

Each comet assay included duplicate slides for positive and negative controls. The positive control was treated with the toxic agent cyclophosphamide (50 µg/mL; C0768-1G, Sigma-Aldrich, Darmstadt, Germany) for 15 min prior to cell deposition on the slides. The negative control consisted of cells from the 2nd or 3rd passage of static cultures, freshly trypsinized and used as the initial inoculum.

### 4.13. Detection of β-Galactosidase in ASCs

To evaluate potential cellular senescence in bioreactor-cultured ASCs (experiments B_4_ and B_5_), the Senescence β-Galactosidase Staining Kit (Cell Signaling, Danvers, MA, USA) was used following the manufacturer’s protocol. Briefly, pre-inoculation cell samples were collected and compared in duplicate with samples taken at the end of each suspension culture (9 days). Cells were seeded onto sterile 20 × 20 mm coverslips placed in the wells of a 6-well plate to allow adhesion.

The following day, the culture medium was removed, the wells were washed once with PBS, and cells were fixed for 15 min using the kit’s fixation solution. After fixation, the wells were washed once with PBS, and 1 mL of β-Galactosidase staining solution at pH 6.0 was added per well. Plates were incubated overnight at 37 °C in a CO_2_-free incubator to maintain the solution pH.

After incubation, coverslips were removed, air-dried at room temperature, and mounted on glass slides with Entellan for observation under a light microscope (Olympus CX31, Tokyo, Japan). β-Galactosidase activity, a marker of cellular senescence, was assessed by the presence and intensity of blue staining in the cytoplasm of fixed cells.

### 4.14. Micronucleus Analysis

To assess genotoxic damage in bioreactor-cultured ASCs, the micronucleus assay was performed following a modified protocol based on Fenech [69]. Cell samples were collected from trypsinized MCs at the end of the experiments (9 days; B_4_ and B_5_). Cells were seeded in quadruplicate onto sterile 20 × 20 mm coverslips placed in the wells of 6-well plates and incubated at 37 °C in a CO_2_ incubator for 24 h to allow adhesion.

Subsequently, cytochalasin B (3 µg/mL; C6762-1MG, Sigma-Aldrich, Darmstadt, Germany) was added to each well to inhibit cytokinesis and cells were incubated for 96 h, covering at least one cell doubling time and increasing the number of binucleated cells. After incubation, the culture medium was removed, and the wells were washed once with PBS, followed by a 10 min incubation in cold hypotonic KCl solution (75 mM) and fixation in pure methanol for 30 min.

After fixation, each well with the coverslip was washed once with PBS, then 300 µL of DAPI solution (300 nM in 1 mL PBS) was added and incubated for 10 min in the dark. The solution was discarded, the wells were washed once with PBS, and coverslips were transferred onto glass slides with a drop of PBS to maintain adhesion and moisture. Slides were examined using a fluorescence microscope at 40× magnification to visualize binucleated cells (Figure 4(c_1_)).

For each coverslip, 500 cells were randomly analyzed to determine the Nuclear Division Index (NDI), according to Equation (12), and 500 binucleated cells were assessed for micronucleus frequency (Figure 4(c_2_,c_3_)), totaling 2000 cells per analysis for each culture.

(12) $$NDI=\frac{\left[M1+2\left(M2\right)+3\left(M3\right)+4\left(M4\right)\right]}{N}$$

where *M*_1_ to *M*_4_ represent the number of cells containing 1, 2, 3, or 4 nuclei, and ***N*** is the total number of cells analyzed per coverslip (=500).

Each micronucleus assay included quadruplicate samples for positive and negative controls. The positive control (C^+^) was treated with the genotoxic agent mitomycin C (0.5 µg/mL of culture medium; M4287-2MG, Sigma-Aldrich, Darmstadt, Germany) for 24 h prior to cytokinesis block with cytochalasin B for 96 h. The negative control (C^−^, 0 h) consisted of untreated cells derived from the initial inoculum of each bioreactor experiment (B_4_ and B_5_), originating from prior static culture proliferation.

### 4.15. Immunophenotypic Characterization

At the conclusion of B_5_ culture, 4 × 10^6^ ASCs were collected for flow cytometry analysis (FACS Calibur, Becton Dickinson, Franklin Lakes, NJ, USA). Cells were labeled with monoclonal antibodies against CD34 (PE, 555822), CD45 (PE, 555483), CD73 (FITC, 561254), CD90 (FITC, 555595), and HLA-DR (PERCP, 347364) (Becton Dickinson Pharmigen, Franklin Lakes, NJ, USA) following the manufacturer’s instructions. Isotype controls were included in all experiments. A minimum of 10,000 events per sample were recorded. Data acquisition and analysis were performed using Cell Quest and Paint Gate software (Becton Dickinson Pharmingen, Franklin Lakes, NJ, USA).

### 4.16. In Vitro Differentiation Analysis

The differentiation potential of ASCs into mesodermal lineages was evaluated using cells collected at the end of experiments B_1_–B_5_. Dissociated cells were seeded in 6-well plates and induced to differentiate following the protocols of StemPro Osteogenic (A10072-01) and Adipogenic (A10070-01) Differentiation Kits (Gibco, New York, NY, USA). Cells were cultured for the period recommended by the manufacturer, and differentiation was assessed via Alizarin Red S staining for osteogenesis and Oil Red O staining for adipogenesis. Images of the cultures were captured using an inverted light microscope (Tucsen, Fuzhou, China) equipped with a camera and analyzed with ISCapture Professional Imaging Software (Tucsen, Fuzhou, China). Negative controls were maintained under identical conditions with regular medium changes, but were cultured only in basal medium (αMEM) supplemented with 10% FBS and 1% AA.

### 4.17. Statistical Analysis

Histograms, proliferation curves, and linear regressions, along with their corresponding coefficients and statistical comparisons, were generated using GraphPad Prism 9 (GraphPad Software, San Diego, CA, USA). Depending on the data distribution, variables were analyzed using a parametric one-way ANOVA followed by Tukey’s post hoc test, or a non-parametric Kruskal–Wallis test followed by Dunn’s post hoc test when Shapiro-Wilk testing indicated non-normality. Comparisons between two independent samples were performed using the Mann–Whitney U test. All results are presented as mean ± standard error of the mean (SEM), with a significance threshold set at *p* < 0.05.

### 4.18. Ethical Considerations

This study was approved by the Research Ethics Committee (REC) of the School of Sciences and Letters of Assis (UNESP) and registered on Plataforma Brasil (CAAE: 58107716.0.0000.5401). All procedures were conducted in compliance with the regulations established by Resolution 466/12 of the National Health Council—Ministry of Health. Donors of adipose tissue were fully informed about the study’s objectives and provided written informed consent prior to tissue collection.

## 5. Conclusions

In conclusion, this study demonstrates that large-scale expansion of ASCs in stirred-tank bioreactors can be effectively optimized through the systematic adjustment of key process parameters, enabling high cell yield, while preserving classical functional attributes, including viability, immunophenotype, and differentiation potential. Importantly, the progressive accumulation of DNA damage and increased senescence markers observed during expansion occurred independently of these conventional performance indicators. This finding reveals a dissociation between proliferation-based metrics and genomic integrity, indicating that standard criteria used for bioprocess optimization may not fully capture relevant biological alterations.

Rather than reflecting an immediate impairment of cell function, these results highlight that genomic integrity represents an additional and independent dimension of cell quality. Therefore, incorporating genotoxic and senescence-associated endpoints into bioprocess evaluation provides a more comprehensive framework for assessing stem cell expansion and may support the development of more robust strategies for large-scale manufacturing of cell-based therapies.

## Figures and Tables

**Table 1:** Fixed and variable parameters evaluated in each single experiment conducted in the stirred-tank bioreactor.

**Fixed Operational Parameters**											
**Cell Type**		**Working Volume**		**Culture Medium Supplementation**		**Culture** **Temperature**		**MC Type and Concentration**		**Aeration Method**	
ASCs at the 2nd or 3rd passage		600 mL of αMEM culture medium		10% FBS and 1% AA		36.5 °C		Cultispher-S^®^ (1 g/L)		Headplate injection	
**Variable operational parameters evaluated in each bioreactor culture**											
**Condition/cultivation period**		**Initial inoculum concentration**		**Cell-to-MC ratio**		**DO concentration**		**pH**		**Agitation speed**	Medium renewal regimen *
		**Cells/mL**	**Total cells**	**Cells/MC**	**Cells/cm^2^**	**Set point**	**Average**	**Set point**	**Average**		
**B_1_**	21 days	4.8 × 10^4^	2.88 × 10^7^	60:1	3.200	20%	19.9%	7.30	7.31	70 rpm	25%/72 h
**B_2_**	21 days	2.4 × 10^4^	1.44 × 10^7^	30:1	1.600	2%	2.1%	7.30	7.31	70 rpm	25%/72 h
**B_3_**	21 days	2.4 × 10^4^	1.44 × 10^7^	30:1	1.600	20%	19.9%	7.30	7.31	50 rpm	25%/72 h
**B_4_**	9 days	1.2 × 10^4^	7.20 × 10^6^	15:1	800	20%	19.8%	7.30	7.32	50 rpm	25%/72 h
**B_5_**	9 days	1.2 × 10^4^	7.20 × 10^6^	15:1	800	20%	19.7%	7.30	7.39	50 rpm	25%/48 h

* Medium renewal strategy was initiated on day 3 of cultivation. DO: dissolved oxygen; MC: microcarrier; FBS: fetal bovine serum; AA: antibiotic–antimycotic solution. Medium renewal regimen corresponds to the percentage of medium replaced at each feeding interval.

**Table 2:** Kinetic and metabolic parameters of ASC cultures measured under different bioreactor conditions (B_1_–B_5_).

**Kinetic** **Parameters**	**Culture Conditions**						
	**B_1 (60:1; 20% DO;_** **_70rpm)_**	**B_2 (30:1; 2% DO; 70rpm)_**		**B_3 (30:1; 20% DO; 50rpm)_**		**B_4 (15:1; 20% DO; 50rpm; Medium renewed every 72h)_**	**B_5 (15:1; 20% DO; 50rpm; Medium renewed every 48h)_**
		**Exponential Phase 1**	**Exponential Phase 2**	**Exponential Phase 1**	**Exponential Phase 2**		
Exponential phase (days)	7–12	2–6	8–12	2–7	10–14	3–7	3–7
R^2^ (exponential phase)	0.9328	0.9661	0.9899	0.8763	0.9412	0.8516	0.9667
Exponential growth equation	y = 0.0027x + 11.24	y = 0.0135x + 9.097	y = 0.0056x + 9.543	y = 0.0144x + 9.413	y = 0.0088x + 9.644	y = 0.0143x + 9.250	y = 0.0191x + 8.811
μmax (growth rate)	0.0027 h^−1^	0.0135 h^−1^	0.0056 h^−1^	0.0144 h^−1^	0.0088 h^−1^	0.0143 h^−1^	0.0191 h^−1^
Doubling time (exponential phase)	256.6 h	51.3 h	123.7 h	48.1 h	78.7 h	48.5 h	36.3 h
Maximum cell productivity	376.98 cells/mL/h (day 21)	125 cells/mL/h (day 19)		751.49 cells/mL/h (day 14)		689.81 cells/mL/h (day 9)	809.52 cells/mL/h (day 7)
Metabolic yield coefficients *	**Nutrient consumption and metabolite production during the exponential growth phase**						
	**B_1_**	**B_2_ (P_1_)**	**B_2_ (P_2_)**	**B_3_ (P_1_)**	**B_3_ (P_2_)**	**B_4_**	**B_5_**
$(Y_{\frac{Glu}{x}}$)	5.11	−1.26	7.17	2.99	1.24	3.86	1.89
$(Y_{\frac{Gln}{x}}$)	0.00107	−0.00066	−0.00018	0.00005	0.00015	0.00057	0.00075
$(Y_{\frac{Lac}{x}}$)	6.92	5.79	3.92	3.01	1.33	4.18	6.88
$(Y_{\frac{Glm}{x}}$)	−0.00003	0.0005	−0.0003	0.00011	0.00006	0.00003	0.00024
$(Y_{\frac{Amn}{x}}$)	0.15	0.177	0.426	−0.076	−0.051	0.059	0.331

* Values expressed as pg/cell.

## Data Availability

The original contributions presented in this study are included in the article. Further inquiries can be directed to the corresponding authors.

## Abbreviations

The following abbreviations are used in this manuscript:

AA	Antibiotic-antimycotic solution
αMEM	α-modified Eagle’s medium
AO/BE	Acridine Orange (3,6-dimethylaminoacridine) combined with Ethidium Bromide (3,8-diamino-5-ethyl-6-phenylphenanthridinium)
ASC(s)	Adipose-derived stem cells
B_1_	Bioreactor experiment with 60 cells per MC, 20% DO and 70 rpm agitation speed
B_2_	Bioreactor experiment with 30 cells per MC, 2% DO and 50 rpm agitation speed
B_3_	Bioreactor experiment with 30 cells per MC, 20% DO and 50 rpm agitation speed
B_4_	Bioreactor experiment with 15 cells per MC, 20% DO, 50 rpm agitation speed, and 25% medium renewal every 72 h
B_5_	Bioreactor experiment with 15 cells per MC, 20% DO, 50 rpm agitation speed, and 25% medium renewal every 48 h
DAPI	4′,6-diamidino-2-phenylindole dihydrochloride
DMSO	Dimethyl sulfoxide
DO	Dissolved oxygen
FBS	Fetal bovine serum
FI	Fold increase
ISCT	International Society for Cellular Therapy
LMP	Low-melting-point
MC(s)	Microcarrier(s)
MSC(s)	Mesenchymal stromal cells
NDI	Nuclear division index
NMP	Normal-melting-point
PBS	Phosphate-buffered saline
OECD	Organization for Economic Co-operation and Development
ROS	Reactive oxygen species
SEM	Standard error of the mean
